# Role of Gut Microbiota and Oxidative Stress in the Progression of Transplant-Related Complications following Hematopoietic Stem Cell Transplantation

**DOI:** 10.1155/2023/3532756

**Published:** 2023-04-18

**Authors:** Mingxuan Chi, Tao Jiang, Xing He, Haoyu Peng, Yunlong Li, Jiong Zhang, Li Wang, Qing Nian, Kuai Ma, Chi Liu

**Affiliations:** ^1^Department of Nephrology, Sichuan Provincial People's Hospital, Sichuan Renal Disease Clinical Research Center, University of Electronic Science and Technology of China, Chengdu, China; ^2^Chinese Academy of Sciences Sichuan Translational Medicine Research Hospital, Chengdu 610072, China; ^3^Department of Hematology, Sichuan Academy of Medical Sciences and Sichuan Provincial People's Hospital, School of Medicine, University of Electronic Science and Technology of China, Chengdu, Sichuan Province 610072, China; ^4^School of Clinical Medicine, Chengdu Medical College, China; ^5^School of Medicine, University of Electronic Science and Technology of China, Chengdu, China; ^6^Department of Urology, Sichuan Cancer Hospital & Institute, Sichuan Cancer Center, School of Medicine, University of Electronic Science and Technology of China, Chengdu, China; ^7^Department of Blood Transfusion, Sichuan Provincial People's Hospital, University of Electronic Science and Technology of China, Chengdu, China; ^8^Department of Nephrology, Osaka University Graduate School of Medicine, Osaka, Japan

## Abstract

Hematopoietic stem cell transplantation (HSCT), also known as bone marrow transplantation, has curative potential for various hematologic malignancies but is associated with risks such as graft-versus-host disease (GvHD), severe bloodstream infection, viral pneumonia, idiopathic pneumonia syndrome (IPS), lung fibrosis, and sinusoidal obstruction syndrome (SOS), which severely deteriorate clinical outcomes and limit the wide application of HSCT. Recent research has provided important insights into the effects of gut microbiota and oxidative stress (OS) on HSCT complications. Therefore, based on recent studies, we describe intestinal dysbiosis and OS in patients with HSCT and review recent molecular findings underlying the causal relationships of gut microbiota, OS, and transplant-related complications, focusing particularly on the involvement of gut microbiota-mediated OS in postengraftment complications. Also, we discuss the use of antioxidative and anti-inflammatory probiotics to manipulate gut microbiota and OS, which have been associated with promising effects in improving HSCT outcomes.

## 1. Introduction

Hematopoietic stem cell transplantation (HSCT) is a potentially life-saving procedure for a multitude of congenital and acquired diseases of the hematopoietic system, including malignancy, severe hematopoietic deficiency, and immune dysfunction [1]. Human hematopoietic stem cells (HSCs) with strong regenerative potential are uniquely implanted into the bone marrow of recipients, providing long-term multilineage hematopoiesis and reconstituting a complete hematopoietic system [2]. Complications after HSCT, including graft-versus-host disease (GvHD), severe bloodstream infection, viral pneumonia, idiopathic pneumonia syndrome (IPS), and sinusoidal obstruction syndrome (SOS) are closely associated with peritransplant morbidity and mortality and severely limit the wide application of HSCT. Despite efforts made in improving transplant outcomes, such as the high resolution of human histocompatibility locus genotyping, prophylactic use of calcineurin inhibitors [3], and infection control using wide-spectrum antibiotics [4], the management of postengraftment complications remains the cornerstone of successful HSCT.

The gut microbiota benefits from the warm nutrient-rich environment of a healthy gut and serves as an important health regulator for hosts. Firmicutes including *Lactobacillus*, *Streptococcus*, *Mycoplasma*, *Clostridium*, and Bacteroidetes comprise 90% of the total gut microbiota. Healthy gut microbiota contributes to intestinal ecosystem homeostasis. Rapid shifts in the composition and function of intestinal microbial communities, known as intestinal dysbiosis, are associated with intestinal barrier disruption and lead to the development of inflammatory [5], cancer [6], metabolic diseases [7], and neurodegenerative diseases [8]. Patients undergoing HSCT display significant changes in the gut microbiota due to the underlying malignancy and exposures to extensive chemotherapy, immunosuppressants, and systemic antibiotics [9]. Due to the clinical significance of gut microbiota, significant interest has emerged to understand the interplay between gut microbiota and HSCT-related complications and reveal the therapeutic value of this interaction.

Reactive oxygen species (ROS) including hydroxyl radicals (OH), superoxide anions, and hydrogen peroxide (H_2_O_2_) are byproducts of oxidative phosphorylation and trigger the activation of cyclooxygenases, nitric oxide (NO) synthase, lipoxygenases, and nicotinamide adenine dinucleotide phosphate (NADPH) oxidase. HSCTs are known to increase the intracellular and extracellular accumulation of ROS, leading to an oxidative stress (OS) status for the occurrence of chemoradiotherapy conditioning and iron overload. Moreover, many recent studies have shown that both commensal and pathogenic bacteria can alter ROS production and promote the progression of neurodegeneration [10], fatty liver disease [11], and diabetes mellitus [12] (Table 1). OS impairs hematopoietic progenitor function and is potentially associated with posttransplant complications, leading to adverse clinical outcomes.

Targeting the OS and gut microbiota may represent an attractive therapeutic avenue for the management of transplant-related complications after HSCT. This review provides an in-depth examination of the crosstalk between OS, the gut microbiota, and transplant-related complications after HSCT. We first briefly reviewed the intestinal dysbiosis and OS in patients who underwent HSCT followed by comprehensive scrutiny of recent molecular findings underlying the causal relationships between gut microbiota, OS, and transplant-related complications, focusing on the gut microbiota-mediated OS involved in postengraftment complications. A better understanding of these relationships in patients with HSCT may allow unraveling the treatment for transplant-related complications by targeting OS and gut microbiota.

## 2. Intestinal Dysbiosis in Patients Undergoing HSCT: Adverse Effect of Conditioning Regimen and Prophylactic Antibiotics

Gut microbiota can promote intestinal homeostasis and protect intestinal integrity by supporting mucosal immunity maturation and preventing (invading) pathogen colonization [13]. Multiple factors can influence the compositional and functional dynamic balance of the intestinal microbiota, resulting in dysbiosis. HSCT recipients are particularly vulnerable to dysbiosis because of their underlying malignancies, long-term hospitalizations, prolonged application of antibiotics, and the use of preparative regimens prior to transplantation [14].

Dysbiosis in HSCT patients commonly manifests as a reduction in gut microbial diversity, diminished strictly anaerobic commensal bacteria, and expansion of pathogenic bacteria. Metagenomic analysis revealed the mean urinary indoxyl sulfate levels that can serve as an indirect marker of bacterial diversity in all patients receiving allo-HSCT dropped from 42.5 ± 11 mmol/L to 11.8 ± 2.8 mmol/L [15]. Intensive chemotherapy and/or radiation preparative regimens are responsible for the expansion of *Lactobacillales* and *Enterobacteriales* and the prominent loss of *Clostridiales* in mice [16]. Patients routinely consume antimicrobials prophylactically to diminish anaerobic bacteria and prevent opportunistic infections in the early posttransplantation period. However, metagenomic analysis of the stool microbiome revealed that microbial composition shifts and diversity loss were more pronounced after extensive antimicrobial exposure in HSCT patients [15]. The drastic loss of diversity in the microbiota is often accompanied by the expansion of a single taxon. *Enterococcus* predominance is more obvious under exposure to antibiotics such as ciprofloxacin and metronidazole [17] with a notable expansion of *E. faecium* and a complementary decrease in Firmicutes and other commensal phyla [15]. Rifaximin is a prophylactic antibiotic that effectively reduces intestinal infections and subsequent acute GvHD [18]. However, new research shows that rifaximin could contribute to microbiome disruption and favor an outbreak of life-threatening *Candida* spp. infections [19]. Microbial SCFAs, including acetate, butyrate, and propionate, are products of carbohydrate fermentation by the anaerobic commensal bacteria (*Clostridia* spp., for instance). SCFAs can preserve intestinal barrier integrity by supporting the functions of intestinal epithelial and goblet cells through coordinated regulation of tight junction proteins. Furthermore, SCFAs can induce tolerance and inhibit inflammatory cascade mediated by inhibiting nuclear factor kappa b (NF-*κ*B) activation in macrophages, inducing colonic regulatory T (Treg) cell expansion, and upregulating gut-homing molecules and forkhead box protein P3 (Foxp3) of Treg cells [20]. The post-HSCT abundance of butyrogenic bacteria (mainly *Clostridia*) in the intestinal microbiota is higher in patients with resistance to lower tract respiratory infections and lower in patients who are susceptible to acute GvHD (aGvHD) [21].

A low diversity of the intestinal microbiota from allo-HSCT recipients was associated with significantly increased mortality (52%) compared with a high diversity of the intestinal microbiota (8%). Microbiota disruption characterized by loss of diversity and single taxa domination is particularly associated with negative outcomes in allo-HSCT recipients. The domination of enterococci in posttransplant stool specimens is positively related to the subsequent development of gastrointestinal GvHD, and the mean proportion of enterococci increased by 53% at the time of active GvHD [15, 22]. Also, intestinal dysbiosis in HSCT patients is correlated with multiple infections including bloodstream infection [23], diarrhea [9], multidrug-resistant organism (MDRO) infection [24], and pulmonary infections [25]. Moreover, a retrospective observational analysis of 541 patients undergoing allo-HSCT identified that the intestinal microbiota could be associated with relapse/progression of disease after allo-HSCT [26].

## 3. Oxidative Stress in Patients Undergoing HSCT

### 3.1. ROS Generation in HSCT Patients: Conditioning Regimens and Iron Overload

Sustained and high-quality transplantation of donor HSCs requires pretransplantation adaptation. Chemotherapy and total-body irradiation are widely used as myeloablative conditioning regimens in patients before HSCT and remove most of the hematopoietic and immune systems of the host. Ionizing radiation can penetrate cells in living organisms and generate ROS via water radiolysis [27]. ROS react rapidly with macromolecules, including proteins, nucleic acids, and lipids, leading to cell damage and apoptotic cell death [28]. Damaged tissues release damage-associated molecular patterns (DAMPs) and initiate acute inflammatory responses through the activation of mitogen-activated protein kinase (MAPK) and nuclear factor kappa-B (NF-*κ*B) signaling cascades. Such pathways are also regarded as the major mediators or inducers of the propagation of radiation-induced bystander effects that induce ROS to increase and replicate irradiation-related DNA damage in nonirradiated cells [29].

Cytostatic agents, including cyclophosphamide (CTX), busulfan, etoposide, melphalan, and carmustine (BCNU), are widely used in HSCT preconditioning to exert their antitumor action and reduce the recurrence rate. However, in recent years, the accumulation of free radicals has been implicated in the administration of cytostatic agents in various categories both *in vitro* and *in vivo*. Bone marrow stromal cells from patients receiving daunorubicin secreted higher levels of H_2_O_2_ than that of healthy control participants, leading to the accumulation of DNA damage in cocultured hematopoietic cells [30]. Chemotherapeutic agents also disrupt redox balance by impairing antioxidant defense (e.g., superoxide dismutase and catalase) in human beings [31]. Plasma levels of vitamin C and catalase, which are powerful antioxidants for scavenging O^2-^, H_2_O_2_, and OH^−^, decline after the application of melphalan and CTX-BCNU-etoposide conditioning regimens [32]. The oxidant effects of CTX are associated with its active metabolites, such as phosphoramide mustard and acrolein, resulting in the accumulation of ROS, which can cause DNA damage and genetic instability, inducing bone marrow suppression [33]. Moreover, chemotherapy with busulfan, BCNU, and cisplatin can cause depletion of plasma glutathione, a nonenzymatic antioxidant [34], and thus amplify oxidative stress.

**Figure 1:**
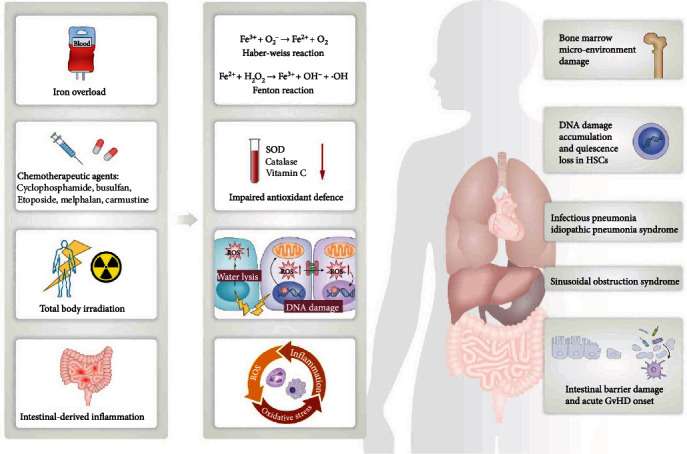
Mechanisms of oxidative stress during HSCT and its impact on the human body. Preengraftment conditioning with chemotherapy and total body irradiation are leading causes of disruption of redox balance and oxidative stress status in patients who underwent HSCT through increasing free radical production and diminishing host antioxidant defense. Iron overload increases ROS production via Haber-Weiss and Fenton reactions. Inflammatory cell infiltration during intestinal inflammation produces excessive oxidative intermediate ROS, which directly damages tissues and further promotes inflammatory response. Oxidative stress status in HSCT patients may contribute to subsequent transplant-related complications including bone marrow microenvironment damage, HSC dysfunction, intestinal barrier damage, and liver and lung injury. HSCT: hematopoietic stem cell transplantation; ROS: reactive oxygen species; HSCs: hematopoietic stem cells.

Iron is a critical cofactor for proteins in the respiratory chain and for cell growth and multiplication. It is potentially toxic to the host when excessive iron is deposited in the cells and tissues of some parenchymal organs. This condition is known as iron overload and is defined by elevated ferritin and liver iron content of approximately 30% and 32%–60%, respectively. Iron overload is a common event associated with HSCT due to the following possible reasons (Figure 1): (1) patients with hematologic diseases usually receive multiple red blood cell transfusions before and after HSCT; (2) chemotherapeutic agents inhibit erythropoiesis, resulting in iron underutilization; (3) the bone marrow, tumor cells, and liver are damaged after high-dose preconditioning, resulting in the release of internal iron pools. The most damaging effect of iron overload is the cycling between Fe^2+^ and Fe^3+^ via Haber-Weiss and Fenton reactions, ultimately generating reactive and toxic free radicals, such as OH^−^ and HO. Iron toxicity induces ROS and triggers inflammation, mediates oxidative and genotoxic stress of HSCs to damage the graft, and promotes recurrence, further damaging the already dysfunctional bone marrow microenvironment of HSCT recipients. Elevated ferritin levels have been associated with decreased overall survival, increased risk of infections, aGvHD, and sinusoidal occlusive disease [35].

### 3.2. Microbiome Changes May Affect ROS Levels in HSCT Patients

OS in HSCT patients induced by pretransplant conditioning and iron overload has been reported in the literature; however, research on the association between microbiota-derived OS and transplant-related complications and outcomes is limited. The clinical significance of gut microbiota-derived OS in a multitude of diseases, including inflammatory, cancer, metabolic, and neurodegenerative diseases, indicates that changes in intestinal homeostasis can extensively influence the OS status in different systems of the body. Specific commensal and pathogenic bacteria can stimulate OS in the intestinal system. Commensal bacteria induce superoxide production by NADPH oxidase-1 and increase cellular ROS by stimulating formyl-peptide receptors on macrophages and neutrophils, resulting in inflammation of the intestinal epithelium [36]. Gut *Lactobacilli* and *Bifidobacterium* can convert nitrate and nitrites into NO, making the gut epithelia a rich source of NO. NO at high concentrations results in a detrimental effect due to the production of ROS, such as superoxide and H_2_O_2_, which further form highly reactive hydroxyl radicals [10]. *E. faecalis* produces substantial extracellular superoxide and derivative reactive nitrogen and oxygen species, such as H_2_O_2_ and OH, through the autoxidation of membrane-associated demethylmenaquinone [37]. However, OS occurring during intestinal instability and inflammation is a risk factor for dysbiosis because it strongly decreases microbial diversity and promotes the expansion of specific bacterial taxa. Leukocyte infiltration accompanied by the generation of reactive oxygen and nitrogen species during intestinal inflammation kills strictly anaerobic bacteria that are susceptible to oxygen intoxication and also promotes the selective growth of bacterial groups including *Enterobacteriaceae* (*Salmonella* and *Citrobacter*) as well as *Escherichia coli* through nitrate and tetrathionate respiration [38, 39].

### 3.3. The Adverse Effects of Excessive ROS and OS Status on HSCT Outcomes

OS is commonly resulting from chronic inflammation and subsequent generation of ROS and nitrogen species that are capable of damaging cellular DNA, protein, and organelles, thus altering gene expression and cell phenotypic traits. OS is suspected to promote cancer and contribute to diverse degenerative neurological disorders, cardiac dysfunction, and aging. The biological characteristics of HSCs are tightly regulated by the OS, and the control of ROS levels is important to maintain their self-renewal capacity. At low concentrations, ROS and reactive nitrogen species control diverse cellular functions, such as stem cell differentiation, and are used in intercellular communication. Murine HSCs with low ROS levels are more quiescent and exhibit increased longitudinal self-renewal and pluripotent differentiation compared to HSCs with higher ROS levels [40]. Exceedingly high ROS levels, which occur during important OS conditions such as chronic inflammation or iron overload, can promote quiescence loss and subsequently limit the capacity for regeneration and reconstitution of the entire hematopoietic system after transplantation into recipients [41, 42]. Excess free radicals and ROS cause severe damage to biological macromolecules (especially DNA damage) and dysregulation of the cell cycle, leading to inflammation and injury to the intestinal epithelium as well as intestinal dysbiosis, which heralds adverse outcomes and is associated with deteriorated overall survival after HSCT [43].

## 4. Gastrointestinal Toxicities and Bloodstream Infection after HSCT

Patients undergoing HSCT and routinely receiving immunosuppressive therapy are at a high risk of catastrophic bloodstream infections (BSIs); such infections are associated with significant morbidity and mortality after HSCT. In a case-cohort study of 16,875 pediatric and adult patients who underwent HSCT, 13% developed BSI due to bacterial translocation across the compromised mucosal barrier [44].

Mucosal barrier injury is also a frequent complication of allo-HSCT and an independent risk factor for the invasion of the gut microbiota into the bloodstream. Healthy intestinal epithelial cells, including intestinal stem cells, goblet cells, and Paneth cells, are connected by tight junctions and assemble into the intestinal epithelium. The intestinal epithelium, with a mucus layer, provides a physical and biochemical barrier, limiting the penetration of microbes and intestinal luminal contents into the host tissues. Pretransplant conditioning with radiation and chemotherapy is associated with increased ROS levels. Excessive OS causes DNA damage, inflammation, and cell apoptosis, leading to shifts in the microbiota, intestinal leakage, and radiation-induced enteritis. Chemoradiation therapy-induced DNA damage promotes the production of epithelial-derived interleukin- (IL-) 1*β*, which initiates intestinal barrier damage by compromising epithelial tight junctions [45]. Patients receiving pretransplant conditioning are not only susceptible to aggravated gastrointestinal epithelial cell damage but also to the elimination of circulating granulocytes and monocytes, markedly increasing susceptibility to subsequent bacterial translocations and disseminated infections [46, 47]. Iron overload is also related to OS status in HSCT patients and can cause tissue damage by protein oxidation, membrane lipid peroxidation, and nucleic acid modification, with the conversion of H_2_O_2_ to ROS [43]. Patients with high pretransplant serum ferritin, a surrogate indicator of tissue iron overload, have an increased incidence of BSI/death (60 vs. 44%, *P* = 0.042) than those with normal levels of pretransplant serum ferritin [35]. The severity of intestinal injury (also referred to as mucositis) after myeloablative conditioning is considered to be the most important determinant of the post-HSCT inflammatory response and is associated with the occurrence of inflammatory complications, including bacteremia, lung injury, and GvHD [48].

*E. coli* and *Klebsiella pneumoniae* BSIs with concomitant gut colonization by these organisms suggest that profound disturbances in the gut microbiota populations play an important role in BSI after HSCT [49]. Furthermore, the dominance of a single bacterial genus such as *Enterococcus* (vancomycin-resistant *Enterococcus* [23]), *Streptococcus* (viridian-group *Streptococcus* [50]), and various Proteobacteria [24] has been identified as the most common cause of bacteremia.

It is imperative to develop strategies to maintain the gut microbiota and gastrointestinal health to prevent subsequent enteric bacterial BSI and improve survival [51]. Prophylactic administration of fluoroquinolones, such as ciprofloxacin and levofloxacin, can reduce the risk of intestinal domination with Gram-negative microbes, including *Proteobacteria* [52] (*Escherichia*, *Klebsiella*, and *Enterobacter*) [53], which are significantly associated with decreased bacteremia without increased risk of *Clostridium difficile*-associated diarrhea, aGVHD, or MDRO [54]. In addition to the prophylactic use of antimicrobial agents, gut decontamination with nonabsorbable antibiotics in the peri-HSCT period was reported to protect against gut-derived BSI by decreasing the microbial load of gut pathogens [55]. For intestinal barrier protection, the IL-1 receptor antagonist anakinra and anti-IL-1*β* antibody canakinumab limit the inflammatory reaction and improve intestinal barrier integrity in HSCT patients and murine [45, 56].

## 5. Graft-Versus-Host Disease after HSCT

### 5.1. Pathophysiology of Acute GvHD (aGvHD)

**Figure 2:**
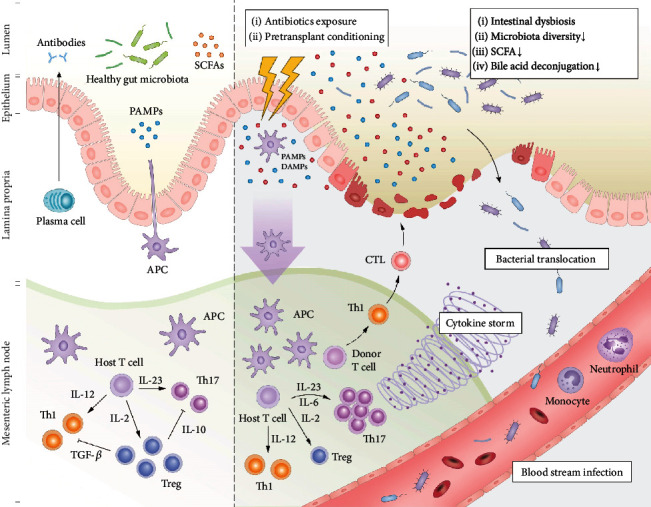
The intestinal damage and the pathogenesis of BSI and GvHD. A healthy intestinal system is important to maintain immune homeostasis. The intestinal homeostasis and epithelial cells are damaged by the cytotoxic conditioning regimen as well as by extensive antibiotic exposure, leading to disruption of the intestinal barrier, intestinal dysbiosis, and decreased production of beneficial metabolites (e.g., SCFAs). Bacteria translocate into circulation, leading to BSI and disseminated infections. DAMPs released by the dying intestinal epithelial cells as well as translocating bacteria and PAMPs activate host APCs resulting in a cytokine storm and donor T cell activation. T cells subsequently proliferate and differentiate into Th1 and Th17 types, which are involved in the activation of CTL that mediate tissue damage. Effector T cells together with cytokine storm attack the epithelial cells of the skin, liver, lung, and gastrointestinal tract, culminating in clinically GvHD. SCFA: short-chain fatty acid; BSI: bloodstream infection; DAMP: danger-associated molecular pattern; PAMP: pathogen-associated molecular pattern; APC: antigen-presenting cell; CTL: cytotoxic T lymphocytes; GVHD: graft-vs.-host disease.

GvHD is a common secondary disease in patients undergoing HSCT, which has long limited the efficacy of HSCT. Before transplantation, the patient's tissues and immune system have been profoundly damaged due to underlying disease, treatment for the disease, infections, and the conditioning regimen. Allogenic T cells from a foreign donor activate and respond upon binding human leukocyte antigens that are expressed on host tissue. A compromised host immune system is incapable of rejecting the immunocompetent cells, leading to amplified CD4^+^/CD8^+^ T cell activation and subsequent GvHD initiation. Subsequently, cytotoxic T lymphocytes (CTLs) and natural killer (NK) cells induce the target cells' apoptosis through the Fas/Fas ligand pathway and perforin/granzyme pathway. Furthermore, inflammatory cytokines synergize with CTLs, resulting in further tissue injury and possible target organ dysfunction (Figure 2). Active GvHD primarily targets the skin (81%), gastrointestinal tract (54%), and liver (50%) of the hosts [57], closely associated with nonrelapse mortality following HSCT.

### 5.2. Intestinal Barrier, Microbial Dysbiosis, and the Onset of aGvHD

Damage to host tissues, especially the intestinal mucosa, caused by the conditioning regimen, is the most important initial step in the pathophysiology of aGvHD. Pretransplant conditioning regimens and GvHD can directly impair gut epithelium, especially Paneth cells. Paneth cell damage contributes to the loss of antimicrobial peptides (e.g., *α*-defensins) and growth factors (e.g., epidermal growth factor and transforming growth factor-*α*), then accelerates the loss of microbial diversity, and compromises epithelial regeneration capacity in GvHD, which leads to a higher risk of nonrelapse mortality [58]. Early studies reported that the GvHD-related mortality was significantly reduced in germ-free mice or when intestinal decontamination was performed [17, 59]. A longitudinal study reported that the development of GvHD was preceded by remarkable shifts in the gut microbiota that can serve as an early predictor of GvHD and transplant-related mortality after HSCT [60], with a predominant role played by Gram-positive bacteria belonging to Firmicutes phylum [61]. The hypothesis that lymphocytes sensitized against microbial antigens cross-react with epithelial antigens in GvHD is the most widely accepted model of microbial interactions in the pathogenesis of GvHD. Microbial products like lipopolysaccharide (LPS) and other pathogen-associated molecular patterns (PAMPs) systemically translocate from the bowel lumen through a damaged intestinal mucosa to the systemic circulation and then stimulate mononuclear cells (monocytes/macrophages) via pathogen recognition receptor (PRR) family such as NOD-like receptors (NLRs) and Toll-like receptors (TLRs) [62]. The amplified activation of these antigen-presenting cells triggers a cytokine storm (tumor necrosis factor-*α* (TNF-*α*) and IL-1) and a lower Treg/T helper (Th) 17 cell ratio, leading to amplification and propagation of a cytokine storm. These cytokines induce inflammatory damage and increase the expression of major histocompatibility complex (MHC) antigens and adhesion molecules in host tissues, enhancing the alloreactivity of mature donor T cells against host tissues, which are equivalent to GvHD [63, 64].

High-throughput metabolomic analysis revealed that GvHD development seems to be associated with major metabolomic changes in the intestinal microbiota compared with patients who did not develop GvHD. AhRs can modulate Th17 response and encourage tolerance by promoting Treg cells [65]. Microbially derived indole compounds are AhR ligands, which show a significant decrease, even undetectable in recipients with GvHD, and are associated with GvHD onset and severity. In addition, reduced plasmalogens, together with increased bile acids and polyunsaturated acids, are potential metabolomic pathways that could be involved in the early proinflammatory response during GvHD [66]. Mucosa-associated invariant T (MAIT) cells are a group of innate-like T cells that inhibit the proliferation of CD4^+^ T cells. Poor reconstitution of MAIT cells after HSCT is significantly associated with the development and severity of GvHD [67]. Peripheral expansion of MAIT cells requires riboflavin (vitamin B2), the metabolite derived from healthy microbiota, which was observed to be significantly decreased in disrupted microbiota of HSCT patients [68]. Intestinal microbial metabolite plasmalogens produced by *Clostridium* strains and *Bifidobacterium longum* have many antioxidant effects in vitro and in vivo [69]. The level of microbiota-derived plasmalogens was dramatically low at the onset of aGvHD, leading to an imbalance between oxidation and antioxidation preceding GvHD. Increasing research on the crosstalk between the host and gut microbiota has provided opportunities to better understand the complex network of GvHD and optimize therapeutic strategies for decreasing HSCT-related morbidity and mortality.

### 5.3. GvHD Treatments Based on Targeting the Gut Microbiota

Prevention of GvHD mainly focuses on T cell depletion and regulation of T cell activation, proliferation, effector, and regulatory functions. Multimodal treatment is often used, but systemic corticosteroids are usually the mainstay of GvHD treatment. From the perspective of gut microbiota, restoring the intestinal epithelium and maintaining intestinal homeostasis represents the adjunct therapeutic strategies to standard immunosuppressive treatment of GvHD without compromising graft-versus-leukemia (GVL) effects. The GVL effect is a type of graft-versus-host reaction targeting leukemic cells in recipients, leading to reduced recurrence and superior survival [70]. Enteral nutrition as first-line nutritional support in patients who undergo HSCT can maintain the intestinal microecology and effectively inhibit GvHD onset [71]. Pretransplant administration of IL-25, a growth factor for goblet cells, allowed the conservation of goblet cells, prevented bacterial translocation, reduced plasma concentrations of interferon-*γ* (IFN-*γ*) and IL-6, and ameliorated GvHD [72]. The glucagon-like peptide 2 promotes the regeneration of Paneth cells and intestinal stem cells, which reduces aGvHD and steroid-refractory GvHD without compromising GVL effects in multiple mouse models [73]. A clinical trial (NCT02641236) revealed a decrease in the incidence of aGvHD in patients who underwent gut decontamination with oral vancomycin and polymyxin B; however, these investigations need to be significantly expanded [55]. Prophylactic administration of antimicrobials is a controversial topic because systemic antibiotic exposure not only suppresses anaerobic bacterial growth but also causes microbial diversity loss, decreases the production of anti-inflammatory SCFAs, and increases the incidence and severity of GvHD [74]. On the other hand, fecal microbiota transplantation (FMT) and probiotic supplementation have been analyzed in clinical trials, with a promising therapeutic value of restoring the intestinal microbiota, diminishing OS, reducing the incidence and severity of GvHD, and preventing drug-resistant bacterial colonization and virus infections [75–77]. The microbe-derived SCFA butyrate and propionate can effectively expand Foxp3^+^ Tregs through upregulation of GPRs expression, thus effectively inhibiting the occurrence of GvHD and promoting immune remodeling [20, 78]. Oral administration of *Bacteroides fragilis* has a beneficial effect on the preservation of intestinal integrity and reduces inflammatory cytokine levels by increasing SCFAs, IL-22, and Treg cells [79].

### 5.4. Oxidative Stress and the Development of aGvHD

Inflammation is a key driver of GvHD; longstanding inflammatory conditions could result in increased oxidative stress. Leukocyte filtration induced by intestinal inflammation results in superoxide production by NADPH oxidase-1, increasing cellular ROS [36]. During an allogeneic immune response, the translocating intestinal flora activates neutrophils, the largest human leukocyte population. The neutrophil infiltration could amplify the tissue damage and contribute to GvHD in the manner of producing ROS. Selective NOX2 deficiency in neutrophils impairing ROS production led to lower levels of tissue damage, GvHD-related mortality, and effector phenotype T cells. *Enterococcus faecalis* is a commensal microorganism of the human intestinal tract that produces substantial extracellular superoxide (O2^−^) and derivative ROS such as H_2_O_2_ and hydroxyl radical, through autoxidation of membrane-associated demethylmenaquinone. The predominance of *Enterococcus faecalis* in GvHD patients was confirmed in metagenomic analysis of fecal microbiome [15]. Excessive ROS produced by *Enterococcus faecalis* could increase DNA damage in colonic epithelial cells and thus may contribute to active GvHD [37, 80].

The levels of NO and its metabolites increase in mice with GvHD, which may play a role in the pathogenetic mechanism of GvHD. Treatment with NO synthesis inhibitor significantly reduces the levels of NO production and bacterial translocation across the intestine, abrogates GvHD-associated enteropathy, and reduces lymphocytic infiltration in the intestinal epithelium, as a result, prolonging the survival of rats with GvHD [81, 82]. As we mentioned above, intestinal injury plays a pivotal role in the development of acute GvHD by providing a portal of entry for Gram-negative bacteria and LPS to enter the host tissues. Ellison et al. reported that LPS injection can consistently induce intestinal epithelial cell apoptosis in graft-versus-host mice triggering mucosal macrophages to release NO, and macrophage-derived NO is the principal mediator of intestinal injury in GvHD [83]. The released NO compromises the integrity of the intestinal epithelium and makes it more permeable to endotoxin. As this occurs, a vicious cycle of intestinal epithelial injury is established in which more endotoxin triggers the release of more NO, and so on [84].

On the other side, oxidative stress can intensify inflammatory responses. Damage of oxidative stress results in oxidized proteins, glycated products, and lipid peroxidation and then turns into the release of inflammatory signal molecules and peroxiredoxin 2 (PRDX2), a ubiquitous redox-active intracellular enzyme [85]. PRDX2 from LPS-stimulated macrophages can alter the redox status of cell surface receptors and allow the induction of inflammatory cascade in chronic inflammatory diseases [85]. Therefore, overproduction of oxidative stress can activate a variety of inflammatory mediators that involve in amplifying the inflammation and form a vicious circle that contributes to the GvHD development in HSCT patients. The strategies to limit oxidative stress in GvHD are highly desirable. Sofi et al. [86] reported that Trx1 is a common antioxidant enzyme that can reduce ROS accumulation in donor T cells and decrease downstream molecules including NF-*κ*B and T-bet, which restrained the ability of T cells to activate, expand, and migrate to the target organs in response to alloantigens in vivo. The administration of human recombinant Trx1 can decrease the pathogenicity of T cells and severity of GvHD and preserve the GVL effect, which has a great translational potential in patients with hematological malignancies undergoing allo-HCT.

## 6. Pulmonary Complications after HSCT

Pulmonary complications (PCs) are reported in up to 70% of HSCT recipients and account for significant morbidity and mortality [25]. HSCT patients are immunocompromised after engraftment as a consequence of chemotherapy, irradiation, acute/chronic GvHD, and maturing recipient marrow. In the postengraftment period, patients are at risk of opportunistic infections by *Pneumocystis jirovecii* and cytomegalovirus. Further, patients represent increased susceptibility to infectious pneumonitis, commonly associated with respiratory viruses, including influenza, respiratory syncytial, and adenoviruses [87]. In addition, chronic GvHD (cGvHD) can also occur later in the postengraftment period where the lung involvement results in chronic obstructive or restrictive pulmonary diseases.

Several studies have reported the relationship between intestinal dysbiosis and many pulmonary diseases, such as allergic airway diseases [88], obstructive pulmonary diseases [89], lung cancer [90], and pneumonia [91]. Therefore, it is pertinent to explore the influence of gut-lung crosstalk on the occurrence of PCs in HSCT recipients. Harris et al. performed a single-center observational study on 94 patients who underwent HSCT and were previously enrolled in a protocol for 16S ribosomal RNA sequencing of the fecal microbiota. They found that low diversity and *γ*-*proteobacteria* dominance in the fecal microbiota (which included common respiratory pathogens) were the independent predictors for the occurrence of PC postengraftment and overall mortality [25]. One possible mechanism is that the impaired gut barrier may facilitate microbial translocation to the lungs through circulation or indirect lung injury by a microbiota-induced systemic inflammatory response, provoking alveolar inflammation and pulmonary dysfunction. Another study analyzing post-HSCT lung microbiota in humans reported that increased relative abundance of *Proteobacteria* in the lung was correlated with impaired lung function after engraftment [92]. These evidences indicate toward a disordered gut-lung axis underlying postengraftment PCs. LPS is a structural component of Gram-negative bacteria and was shown to cause innate immune activation, accumulation of alloreactive T cells, and histologic damage by interacting with TLR4 in allo-HSCT models. Treatment with a TLR4 antagonist could protest against transplant-related lung injuries after HSCT [93]. This research confirmed the role of LPS in promoting the development of alloimmune lung injury after HSCT independent from systemic GvHD in the allo-HSCT model without systemic GvHD. On the other side, the microbial-derived metabolites of SCFAs have the ability to modulate host inflammation and promote immune tolerance against various bacterial and viral infections. HSCT patients with higher levels of SCFA-producing microbial communities were fivefold less likely to develop the pulmonary virus infection with lower respiratory tract infection, independent of other factors (adjusted HR = 0.22, 95% CI 0.04-0.69) [94]. Restoring the balance of endogenous gut microflora may play a role in the treatment of postengraftment PCs by elevating SCFA production.

OS may also play an important role in the pathogenesis of lung injuries, such as IPS and lung fibrosis, following HSCT. The lung is especially susceptible to oxidative damage because it has the largest endothelial surface area in the body, making it vulnerable to circulating toxins. Gut *Lactobacilli* and *Bifidobacterium* possess the ability to convert nitrate and nitrites into NO, making the gut epithelia a rich source of NO [95]. Similarly, *Streptococcus* and *Bacillus* produce NO from L-arginine using nitric oxide synthase. A higher pulmonary concentration of NO combined with superoxide results in the formation of peroxynitrite, a strong oxidant that can oxidize a number of biomolecules including tyrosine-containing proteins, resulting in nitrotyrosine formation. An increased concentration of exhaled NO in the lower respiratory tract and increased nitrotyrosine formation in the alveolar fluid following HSCT were identified as potential markers of IPS [96]. IPS is characterized by noninfectious diffuse lung injury associated with a high-dose chemotherapy regimen (BCNU, cyclophosphamide, and cisplatin) and the incidence of GvHD after HSCT. Murine models of IPS have shown that the conditioning regimen causes lung injury beginning with substantial OS, which further promotes intense monocytic cellular infiltration and macrophage activation. An increased alveolar macrophage population in the epithelial lining fluid has a significantly higher oxidative burst, which may further exacerbate lung inflammation and widespread alveolar injury [97]. In addition, increased ROS and cellular DNA damage in pulmonary fibroblasts are key events in the progression of pulmonary fibrosis, which is frequent post-HSCT [98].

## 7. Sinusoidal Obstruction Syndrome

Hepatic SOS, also known as venoocclusive disease, is a potentially life-threatening complication that occurs in 13% of HSCT patients, belonging to a group of diseases increasingly identified as transplant-related, systemic endothelial diseases [99]. Severe SOS results in multiorgan dysfunction with a mortality rate > 80%. The SOS primarily insults both sinusoidal endothelial cells and hepatocytes in zone 3 of the hepatic acinus, which can be triggered by multiple factors including the toxicity of the conditioning regimens [100], cytokine cascade, microbial endotoxins, immune and alloreactivity.

Elevated oxidative stress in HSCT patients may be involved in the development of SOS. (-)-Epicatechin is a natural flavonol that was found to obviously enhance liver GSH levels and reduce the increased ROS amounts, thus reversing liver oxidative injury and attenuating SOS by activating nuclear translocation of nuclear factor erythroid 2-related factor 2 (Nrf2) antioxidant pathway [101]. As mentioned above, iron accumulation promotes the production of ROS via the catalytic activity of free iron. As the liver is one of the organs in which iron preferentially accumulates, oxidative stress promoted by iron overload in livers after conditioning regimens might be attributed to triggering and exacerbating hepatic injury including SOS in HSCT patients [102]. Yeom et al. demonstrated that ROS levels of the murine liver increased according to cumulative iron dose and correlations with pathologic score for SOS, including sinusoidal hemorrhage and endothelial damage, in HSCT mice with no significant differences between the syngeneic and allogeneic groups [103]. Mitigating oxidative stress with antioxidants has shown protective effects on SOS-related liver injury in many studies [101, 104, 105]. Sesame oil has antioxidant properties that offer better protection against increased blood pressure, hyperlipidemia, and lipid peroxidation by increasing enzymatic and nonenzymatic antioxidants. Inhibiting OS with prophylactic sesame oil prevents the rounding up of sinusoidal endothelial cells and thus attenuates SOS in murine [105].

Preclinical studies suggest that microbial products translocated across impaired intestinal barriers may participate in the pathogenesis of endothelial damage, interfering with procoagulant and fibrinolytic endothelial responses [106]. LPS in especially activates various signaling mechanisms in endothelial cells, ultimately leading to cellular dysfunction and injury [107]. A retrospective case-control study in allo-HSCT pediatric patients conducted by Masetti et al. reported that having healthy gut microbiota characterized by a high diversity and richness of beneficial microorganisms in the pretransplant period is associated with a reduced occurrence of SOS. The disrupted intestinal barrier with depleted beneficial taxa and low production of beneficial SCFAs could lead to greater translocation of microbial molecules. The microbial endotoxin, particularly LPS, translocates across impaired intestinal barriers, reaches the liver sinusoid through the portal vein, and participates in endothelial damage by activating various signaling mechanisms, including NF-*κ*B and p38 MAPK [107, 108]. LPS-induced nitrooxidative stress may also participate in damaging liver microcirculation. The iNOS expression was increased in livers of the LPS-injected mouse group, evidenced by increased liver dihydroethidium staining and increased liver protein nitrotyrosination which can be blunted by the effect of iNOS inhibition [109]. These endothelial changes lead to the narrowing of the central vein lumen and obstruction of the blood flow. This is followed by the organization of subintimal edema and deposition of additional collagen. Thickened collagen cuffs surrounding the central veins characterize chronic SOS.

## 8. Conclusion

**Figure 3:**
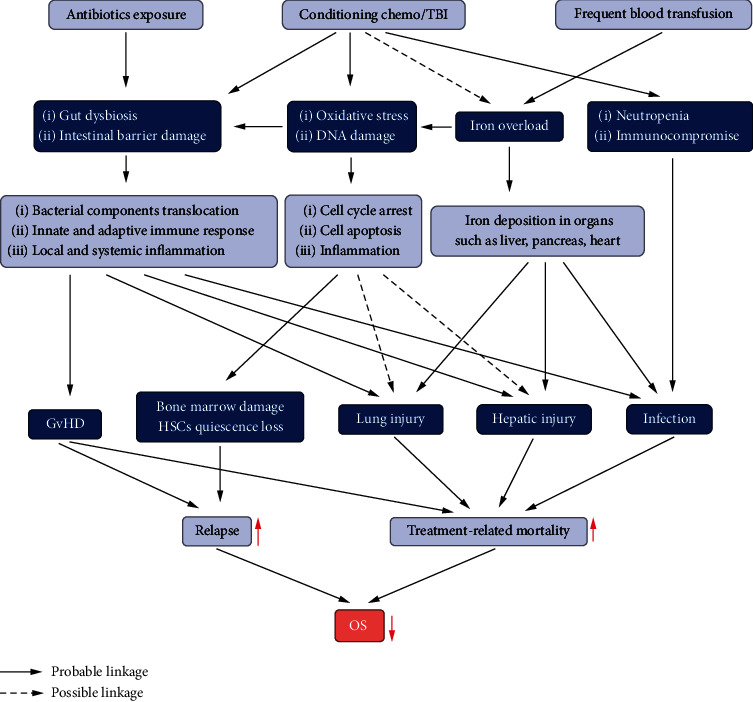
The interplay between intestinal dysbiosis and oxidative stress as well as the transplant-related complications. The interaction between intestinal dysbiosis and oxidative stress caused by preengraftment conditioning and prophylactic antibiotics exposure in this picture. These pathogeneses contribute to different HSCT-related complications such as GvHD, BSI, HSC dysfunction, pulmonary/hepatic injury, and infections which are leading cause of relapse and treatment-related mortality that affect the OS of HSCT. HSCT: hematopoietic stem cell transplantation; GVHD: graft-vs.-host disease; BSI: bloodstream infection; HSCs: hematopoietic stem cells; OS: overall survival.

Intestinal dysbiosis and OS caused by preengraftment conditioning and prophylactic antibiotics result in different HSCT-related complications such as BSI, GvHD, pulmonary injury, and hepatic injury, which are the leading causes of adverse outcomes after HSCT. Disturbance in intestinal microbiota is due to the conditioning regimen, antimicrobial administration, and iatrogenic immunocompromisation in patients undergoing HSCT. Preengraftment conditioning affects the intestinal mucosa due to increased OS and DNA damage in the intestinal epithelial cells. Translocation of commensal and pathogenic bacteria into the bloodstream through impaired intestinal barriers may induce BSIs and host immune responses. Excessive translocation of microbial components leads to allogeneic donor T cell activation and a series of cytokine storms, greatly enhancing the immune response to the recipient antigen and launching cytotoxic attacks on the recipient target cells, which are positively related to the GvHD occurrence. Gut bacteria and their endotoxins can cause pulmonary and liver inflammation and infection through hematogenous dissemination and are also related to pulmonary infections, IPS, and SOS posttransplantation. Several studies have reported antibiotic-mediated decrease in gut bacterial diversity. Further, strategies are also described for restoring the intestinal flora using fecal microbial transfer and probiotics in an aim to manage transplant-related complications and improve clinical outcomes (Figure 3). The immunoregulatory effects of microbial metabolites on SCFAs have also been confirmed in GvHD. Removing the disturbance of redox balance to antioxidant supplements and OS depletion by reducing preconditioning intensity and decreasing iron accumulation has beneficial effects in the management of GvHD, infections, and organ injury in HSCT patients. However, further studies are needed to elucidate the role of intestinal flora-mediated OS in the pathology and treatment of HSCT-related complications, which may provide additional understanding of the pathways employed by gut microbiota in mediating the process of HSCT-related complications.

## Figures and Tables

**Table 1:** Role of gut microbiota-derived oxidative stress in the progressions of different diseases.

Intestinal microbiota	Mechanisms	Relative diseases	Reference
*Enterococci faecalis* ↑	Increase the production of hydroxyl radicals, contribute to DNA breaks, point mutations, and protein-DNA crosslinking, and induce aneuploidy in colonic epithelial cells	Colorectal cancer	[110]
*Proteobacteria* ↑ *Bifidobacteria* ↓	Contributes to the occurrence of dementia not only through the significant reduction of beneficial SCFAs but also through interfering with lipid metabolism	Alzheimer's disease	[111]
Gut-lung axis	Activating oxidative stress through TLR4/NF-kB pathway in the lung and mediating lung injury through the regulation of the gut barrier	Acute lung injury	[112]
Butyrate producers ↓: *Fusobacterium* *Veillonella* *Atopobium parvulum*	Dysbiosis dampen host H_2_S defense systems induce mitochondrial dysfunction likely resulting in ROS production, contributing to mucus degradation, opening the intestinal barrier to toxic compounds and pathobionts	Crohn's disease	[113]
*Prevotella* *Clostridium*	Produce endogenous H_2_, which have antioxidant properties to neutralize toxic hydroxyl radicals, downregulate the expression of proinflammatory factors, and preserve cerebrovascular reactivity	Parkinson's disease	[114]
*Escherichia coli* ↑	Increase production of uric acid, which contributes to the overproduction of oxygen free radicals, vascular endothelial dysfunction, and inflammation	Atherosclerosis	[115]
*Eggerthella lenta* ↑ *Fusobacterium nucleatum* ↑	Increase serum uraemic toxins, which are relative to increased severity of oxidative stress, glomerulosclerosis, and renal fibrosis and increased serum levels of creatinine and/or urea in sham-fed rats	End-stage renal disease	[116]
*H. pylori*	Produce and induce the production of ROS by neutrophils and macrophages	—	[117]
*Lactobacilli* *Bifidobacteria*	High catalase and *α*,*α*-diphenyl-*β*-picrylhydrazyl free radical scavenging activity	Anticancer effect	[118]
*Lactobacillus rhamnosus* GG	Ameliorates alcohol-induced intestinal oxidative stress, intestinal hyperpermeability, and liver injury in rodent models of alcohol steatohepatitis	Alcoholic liver disease	[119]

## Abbreviations

HSCT: Hematopoietic stem cell transplantation
HSCs: Hematopoietic stem cells
GvHD: Graft-versus-host disease
IPS: Idiopathic pneumonia syndrome
SOS: Sinusoidal obstruction syndrome
ROS: Reactive oxygen species
OH: Hydroxyl radical
H_2_O_2_: Hydrogen peroxide
NO: Nitric oxide
OS: Oxidative stress
SCFAs: Short-chain fatty acids
AhR: Aryl hydrocarbon receptor
Treg: Regulatory T
Foxp3: Forkhead box protein P3
MDRO: Multidrug-resistant organisms
DAMPs: Damage-associated molecular patterns
MAPK: Mitogen-activated protein kinase
NF-*κ*B: Nuclear factor kappa-B
CTX: Cyclophosphamide
BCNU: Carmustine
BSI: Bloodstream infection
IL-1*β*: Interleukin-1*β*
PAMPs: Pathogen-associated molecular patterns
GPRs: G protein-coupled receptors
TNF: Tumor necrosis factor
Th: T helper
MHC: Major histocompatibility complex
MAIT: Mucosa-associated invariant T
GVL: Graft-versus-leukemia
FMT: Fecal microbiota transplantation
PCs: Pulmonary complications
LPS: Lipopolysaccharide.

## References

[1] Barriga F., Ramirez P., Wietstruck A., Rojas N. (2012). Hematopoietic stem cell transplantation: clinical use and perspectives. *Biological Research*.

[2] Copelan E. A. (2006). Hematopoietic stem-cell transplantation. *The New England Journal of Medicine*.

[3] Choi S. W., Levine J. E., Ferrara J. L. (2010). Pathogenesis and management of graft-versus-host disease. *Immunology and Allergy Clinics of North America*.

[4] Tomblyn M., Chiller T., Einsele H. (2009). Guidelines for preventing infectious complications among hematopoietic cell transplantation recipients: a global perspective. *Biology of Blood and Marrow Transplantation*.

[5] Amoroso C., Perillo F., Strati F., Fantini M. C., Caprioli F., Facciotti F. (2020). The role of gut microbiota biomodulators on mucosal immunity and intestinal inflammation. *Cell*.

[6] Garrett W. S. (2015). Cancer and the microbiota. *Science*.

[7] Dabke K., Hendrick G., Devkota S. (2019). The gut microbiome and metabolic syndrome. *The Journal of Clinical Investigation*.

[8] Jiang C., Li G., Huang P., Liu Z., Zhao B. (2017). The gut microbiota and Alzheimer's disease. *Journal of Alzheimer's Disease*.

[9] Jabr R., El Atrouni W., Shune L. (2021). *Clostridioides difficile* infection and risk of acute graft-versus-host disease among allogeneic hematopoietic stem cell transplantation recipients. *Transplantation and Cellular Therapy*.

[10] Shandilya S., Kumar S., Kumar Jha N., Kumar Kesari K., Ruokolainen J. (2022). Interplay of gut microbiota and oxidative stress: perspective on neurodegeneration and neuroprotection. *Journal of Advanced Research*.

[11] Borrelli A., Bonelli P., Tuccillo F. M. (2018). Role of gut microbiota and oxidative stress in the progression of non-alcoholic fatty liver disease to hepatocarcinoma: current and innovative therapeutic approaches. *Redox Biology*.

[12] Yuan T., Yang T., Chen H. (2019). New insights into oxidative stress and inflammation during diabetes mellitus-accelerated atherosclerosis. *Redox Biology*.

[13] Thaiss C. A., Zmora N., Levy M., Elinav E. (2016). The microbiome and innate immunity. *Nature*.

[14] Raghunathan V. M., Sheng I., Lim S. H. (2016). Intestinal dysbiosis and allogeneic hematopoietic progenitor cell transplantation. *Journal of Translational Medicine*.

[15] Holler E., Butzhammer P., Schmid K. (2014). Metagenomic analysis of the stool microbiome in patients receiving allogeneic stem cell transplantation: loss of diversity is associated with use of systemic antibiotics and more pronounced in gastrointestinal graft-versus-host disease. *Biology of Blood and Marrow Transplantation*.

[16] Jenq R. R., Ubeda C., Taur Y. (2012). Regulation of intestinal inflammation by microbiota following allogeneic bone marrow transplantation. *The Journal of Experimental Medicine*.

[17] Beelen D. W., Elmaagacli A., Muller K. D., Hirche H., Schaefer U. W. (1999). Influence of intestinal bacterial decontamination using metronidazole and ciprofloxacin or ciprofloxacin alone on the development of acute graft-versus-host disease after marrow transplantation in patients with hematologic malignancies: final results and long-term follow-up of an open-label prospective randomized trial. *Blood*.

[18] Weber D., Oefner P. J., Dettmer K. (2016). Rifaximin preserves intestinal microbiota balance in patients undergoing allogeneic stem cell transplantation. *Bone Marrow Transplantation*.

[19] Marzuttini F., Mancusi A., Bonato S. (2021). Rifaximin use favoured micafungin-resistant Candida spp. infections in recipients of allogeneic hematopoietic cell transplantation. *Annals of Hematology*.

[20] Ghimire S., Weber D., Hippe K. (2021). GPR expression in intestinal biopsies from SCT patients is upregulated in GvHD and is suppressed by broad-spectrum antibiotics. *Frontiers in Immunology*.

[21] Romick-Rosendale L. E., Haslam D. B., Lane A. (2018). Antibiotic exposure and reduced short chain fatty acid production after hematopoietic stem cell transplant. *Biology of Blood and Marrow Transplantation*.

[22] Ingham A. C., Kielsen K., Mordhorst H. (2021). Microbiota long-term dynamics and prediction of acute graft-versus-host disease in pediatric allogeneic stem cell transplantation. *Microbiome*.

[23] Ubeda C., Taur Y., Jenq R. R. (2010). Vancomycin-resistant Enterococcus domination of intestinal microbiota is enabled by antibiotic treatment in mice and precedes bloodstream invasion in humans. *The Journal of Clinical Investigation*.

[24] Kaundal S., Jandial A., Singh H. (2021). Impact of broad-spectrum antibiotic exposures and multidrug-resistant gram-negative bacteremia on hematopoietic cell transplantation outcomes. *Transplant Infectious Disease*.

[25] Harris B., Morjaria S. M., Littmann E. R. (2016). Gut microbiota predict pulmonary infiltrates after allogeneic hematopoietic cell transplantation. *American Journal of Respiratory and Critical Care Medicine*.

[26] Peled J. U., Devlin S. M., Staffas A. (2017). Intestinal microbiota and relapse after hematopoietic-cell transplantation. *Journal of Clinical Oncology*.

[27] Santacruz-Gomez K., Sarabia-Sainz A., Acosta-Elias M. (2018). Antioxidant activity of hydrated carboxylated nanodiamonds and its influence on water *γ*-radiolysis. *Nanotechnology*.

[28] Buonanno M., de Toledo S. M., Pain D., Azzam E. I. (2011). Long-term consequences of radiation-induced bystander effects depend on radiation quality and dose and correlate with oxidative stress. *Radiation Research*.

[29] Klammer H., Mladenov E., Li F., Iliakis G. (2015). Bystander effects as manifestation of intercellular communication of DNA damage and of the cellular oxidative status. *Cancer Letters*.

[30] Li Y., Xue Z., Dong X. (2020). Mitochondrial dysfunction and oxidative stress in bone marrow stromal cells induced by daunorubicin leads to DNA damage in hematopoietic cells. *Free Radical Biology & Medicine*.

[31] dos Santos T. N., Duarte F. B., Filho P. A. M. (2016). Association of oxidative stress and DNA damage with grafting time in patients with multiple myeloma and lymphoma submitted to autologous hematopoietic stem cell transplantation. *Revista da Associação Médica Brasileira*.

[32] Goncalves T. L., Benvegnu D. M., Bonfanti G., Frediani A. V., Rocha J. B. (2009). *δ*-Aminolevulinate dehydratase activity and oxidative stress during melphalan and cyclophosphamide-BCNU-etoposide (CBV) conditioning regimens in autologous bone marrow transplantation patients. *Pharmacological Research*.

[33] Deng J., Zhong Y. F., Wu Y. P. (2018). Carnosine attenuates cyclophosphamide-induced bone marrow suppression by reducing oxidative DNA damage. *Redox Biology*.

[34] Jonas C. R., Puckett A. B., Jones D. P. (2000). Plasma antioxidant status after high-dose chemotherapy: a randomized trial of parenteral nutrition in bone marrow transplantation patients. *The American Journal of Clinical Nutrition*.

[35] Pullarkat V., Blanchard S., Tegtmeier B. (2008). Iron overload adversely affects outcome of allogeneic hematopoietic cell transplantation. *Bone Marrow Transplantation*.

[36] Migeotte I., Communi D., Parmentier M. (2006). Formyl peptide receptors: a promiscuous subfamily of G protein-coupled receptors controlling immune responses. *Cytokine & Growth Factor Reviews*.

[37] Huycke M. M., Abrams V., Moore D. R. (2002). Enterococcus faecalis produces extracellular superoxide and hydrogen peroxide that damages colonic epithelial cell DNA. *Carcinogenesis*.

[38] Winter S. E., Thiennimitr P., Winter M. G. (2010). Gut inflammation provides a respiratory electron acceptor for Salmonella. *Nature*.

[39] Weiss G. A., Hennet T. (2017). Mechanisms and consequences of intestinal dysbiosis. *Cellular and Molecular Life Sciences*.

[40] Mantel C. R., O'Leary H. A., Chitteti B. R. (2015). Enhancing hematopoietic stem cell transplantation efficacy by mitigating oxygen shock. *Cell*.

[41] Woolthuis C. M., Brouwers-Vos A. Z., Huls G., de Wolf J. T., Schuringa J. J., Vellenga E. (2013). Loss of quiescence and impaired function of CD34^+^/CD38^low^ cells one year following autologous stem cell transplantation. *Haematologica*.

[42] Khatri R., Krishnan S., Roy S., Chattopadhyay S., Kumar V., Mukhopadhyay A. (2016). Reactive oxygen species limit the ability of bone marrow stromal cells to support hematopoietic reconstitution in aging mice. *Stem Cells and Development*.

[43] Yegin Z. A., Pasaoglu H., Aki S. Z. (2011). Pro-oxidative/antioxidative imbalance: a key indicator of adverse outcome in hematopoietic stem cell transplantation. *International Journal of Laboratory Hematology*.

[44] Dandoy C. E., Kim S., Chen M. (2020). Incidence, risk factors, and outcomes of patients who develop mucosal barrier injury-laboratory confirmed bloodstream infections in the first 100 days after allogeneic hematopoietic stem cell transplant. *JAMA Network Open*.

[45] Kanarek N., Grivennikov S. I., Leshets M. (2014). Critical role for IL-1*β* in DNA damage-induced mucositis. *Proceedings of the National Academy of Sciences of the United States of America*.

[46] Kharya G., Bakane A., Agarwal S., Rauthan A. (2021). Pre-transplant myeloid and immune suppression, upfront plerixafor mobilization and post-transplant cyclophosphamide: novel strategy for haploidentical transplant in sickle cell disease. *Bone Marrow Transplantation*.

[47] Reyes-Gibby C. C., Melkonian S. C., Wang J. (2017). Identifying novel genes and biological processes relevant to the development of cancer therapy-induced mucositis: an informative gene network analysis. *PLoS One*.

[48] van der Velden W. J., Herbers A. H., Feuth T., Schaap N. P., Donnelly J. P., Blijlevens N. M. (2010). Intestinal damage determines the inflammatory response and early complications in patients receiving conditioning for a stem cell transplantation. *PLoS One*.

[49] Tamburini F. B., Andermann T. M., Tkachenko E., Senchyna F., Banaei N., Bhatt A. S. (2018). Precision identification of diverse bloodstream pathogens in the gut microbiome. *Nature Medicine*.

[50] Rashidi A., Kaiser T., Graiziger C. (2020). Specific gut microbiota changes heralding bloodstream infection and neutropenic fever during intensive chemotherapy. *Leukemia*.

[51] Satwani P., Freedman J. L., Chaudhury S. (2017). A multicenter study of bacterial blood stream infections in pediatric allogeneic hematopoietic cell transplantation recipients: the role of acute gastrointestinal graft-versus-host disease. *Biology of Blood and Marrow Transplantation*.

[52] Taur Y., Xavier J. B., Lipuma L. (2012). Intestinal domination and the risk of bacteremia in patients undergoing allogeneic hematopoietic stem cell transplantation. *Clinical Infectious Diseases*.

[53] Stoma I., Littmann E. R., Peled J. U. (2021). Compositional flux within the intestinal microbiota and risk for bloodstream infection with gram-negative bacteria. *Clinical Infectious Diseases*.

[54] Gardner J. C., Courter J. D., Dandoy C. E., Davies S. M., Teusink-Cross A. (2022). Safety and efficacy of prophylactic levofloxacin in pediatric and adult hematopoietic stem cell transplantation patients. *Transplantation and Cellular Therapy*.

[55] Severyn C. J., Siranosian B. A., Kong S. T. (2022). Microbiota dynamics in a randomized trial of gut decontamination during allogeneic hematopoietic cell transplantation. *JCI Insight*.

[56] de Mooij C. E. M., van Groningen L. F. J., de Haan A. F. J. (2020). Anakinra: efficacy in the management of fever during neutropenia and mucositis in autologous stem cell transplantation (AFFECT-2)-study protocol for a multicenter randomized double-blind placebo-controlled trial. *Trials*.

[57] Martin P. J., Schoch G., Fisher L. (1990). A retrospective analysis of therapy for acute graft-versus-host disease: initial treatment. *Blood*.

[58] Levine J. E., Huber E., Hammer S. T. (2013). Low Paneth cell numbers at onset of gastrointestinal graft-versus-host disease identify patients at high risk for nonrelapse mortality. *Blood*.

[59] Jones J. M., Wilson R., Bealmear P. M. (1971). Mortality and gross pathology of secondary disease in germfree mouse radiation chimeras. *Radiation Research*.

[60] Beckman M. F., Morton D. S., Bahrani Mougeot F., Mougeot J. C. (2021). Allogenic stem cell transplant-associated acute graft versus host disease: a computational drug discovery text mining approach using oral and gut microbiome signatures. *Supportive Care in Cancer*.

[61] Greco R., Nitti R., Mancini N. (2021). Microbiome markers are early predictors of acute GVHD in allogeneic hematopoietic stem cell transplant recipients. *Blood*.

[62] Ghimire S., Weber D., Mavin E., Wang X. N., Dickinson A. M., Holler E. (2017). Pathophysiology of GvHD and other HSCT-related major complications. *Frontiers in Immunology*.

[63] Shono Y., Docampo M. D., Peled J. U., Perobelli S. M., Jenq R. R. (2015). Intestinal microbiota-related effects on graft-versus-host disease. *International Journal of Hematology*.

[64] Han L., Zhang H., Chen S. (2019). Intestinal microbiota can predict acute graft-versus-host disease following allogeneic hematopoietic stem cell transplantation. *Biology of Blood and Marrow Transplantation*.

[65] Gutierrez-Vazquez C., Quintana F. J. (2018). Regulation of the immune response by the aryl hydrocarbon receptor. *Immunity*.

[66] Michonneau D., Latis E., Curis E. (2019). Metabolomics analysis of human acute graft-versus-host disease reveals changes in host and microbiota-derived metabolites. *Nature Communications*.

[67] Konuma T., Kohara C., Watanabe E. (2020). Reconstitution of circulating mucosal-associated invariant T cells after allogeneic hematopoietic cell transplantation: its association with the riboflavin synthetic pathway of gut microbiota in cord blood transplant recipients. *Journal of Immunology*.

[68] Gao M. G., Hong Y., Zhao X. Y. (2021). The potential roles of mucosa-associated invariant T cells in the pathogenesis of gut graft-versus-host disease after hematopoietic stem cell transplantation. *Frontiers in Immunology*.

[69] Mawatari S., Sasuga Y., Morisaki T., Okubo M., Emura T., Fujino T. (2020). Identification of plasmalogens in *Bifidobacterium longum*, but not in *Bifidobacterium animalis*. *Scientific Reports*.

[70] Weiden P. L., Sullivan K. M., Flournoy N., Storb R., Thomas E. D., The Seattle Marrow Transplant Team (1981). Antileukemic effect of chronic graft-versus-host disease: contribution to improved survival after allogeneic marrow transplantation. *The New England Journal of Medicine*.

[71] Zama D., Gori D., Muratore E. (2021). Enteral versus parenteral nutrition as nutritional support after allogeneic hematopoietic stem cell transplantation: a systematic review and meta-analysis. *Transplantation and Cellular Therapy*.

[72] Ara T., Hashimoto D., Hayase E. (2020). Intestinal goblet cells protect against GVHD after allogeneic stem cell transplantation via Lypd 8. *Science Translational Medicine*.

[73] Norona J., Apostolova P., Schmidt D. (2020). Glucagon-like peptide 2 for intestinal stem cell and Paneth cell repair during graft-versus-host disease in mice and humans. *Blood*.

[74] Hidaka D., Hayase E., Shiratori S. (2018). The association between the incidence of intestinal graft-vs-host disease and antibiotic use after allogeneic hematopoietic stem cell transplantation. *Clinical Transplantation*.

[75] Qi L., Huang X., He C., Ji D., Li F. (2021). Steroid-resistant intestinal aGVHD and refractory CMV and EBV infections complicated by haplo-HSCT were successfully rescued by FMT and CTL infusion. *The Journal of International Medical Research*.

[76] Biernat M. M., Urbaniak-Kujda D., Dybko J., Kapelko-Slowik K., Prajs I., Wrobel T. (2020). Fecal microbiota transplantation in the treatment of intestinal steroid-resistant graft-versus-host disease: two case reports and a review of the literature. *The Journal of International Medical Research*.

[77] Su F., Luo Y., Yu J. (2021). Tandem fecal microbiota transplantation cycles in an allogeneic hematopoietic stem cell transplant recipient targeting carbapenem-resistant Enterobacteriaceae colonization: a case report and literature review. *European Journal of Medical Research*.

[78] Markey K. A., Schluter J., Gomes A. L. C. (2020). The microbe-derived short-chain fatty acids butyrate and propionate are associated with protection from chronic GVHD. *Blood*.

[79] Sofi M. H., Wu Y., Ticer T. (2021). A single strain of Bacteroides fragilis protects gut integrity and reduces GVHD. *JCI Insight*.

[80] Friedman G., Stepensky P., Abu Ahmad W. (2020). Enterococcal bacteremia in children with malignancies and following hematopoietic stem cell transplantation: a 15-year single-center experience. *The Pediatric Infectious Disease Journal*.

[81] Garside P., Hutton A. K., Severn A., Liew F. Y., Mowat A. M. (1992). Nitric oxide mediates intestinal pathology in graft-vs.-host disease. *European Journal of Immunology*.

[82] Langrehr J. M., Machens C., Zill E. (2000). Bacterial translocation during graft-versus-host disease after small bowel transplantation is reduced following inhibition of inducible nitric oxide synthesis. *Transplantation*.

[83] Ellison C. A., Natuik S. A., McIntosh A. R., Scully S. A., Danilenko D. M., Gartner J. G. (2003). The role of interferon-gamma, nitric oxide and lipopolysaccharide in intestinal graft-versus-host disease developing in F1-hybrid mice. *Immunology*.

[84] Azar Y., Shainer R., Almogi-Hazan O. (2013). Preimplantation factor reduces graft-versus-host disease by regulating immune response and lowering oxidative stress (murine model). *Biology of Blood and Marrow Transplantation*.

[85] Salzano S., Checconi P., Hanschmann E. M. (2014). Linkage of inflammation and oxidative stress via release of glutathionylated peroxiredoxin-2, which acts as a danger signal. *Proceedings of the National Academy of Sciences of the United States of America*.

[86] Sofi M. H., Wu Y., Schutt S. D. (2019). Thioredoxin-1 confines T cell alloresponse and pathogenicity in graft-versus-host disease. *The Journal of Clinical Investigation*.

[87] Peck A. J., Englund J. A., Kuypers J. (2007). Respiratory virus infection among hematopoietic cell transplant recipients: evidence for asymptomatic parainfluenza virus infection. *Blood*.

[88] Hufnagl K., Pali-Scholl I., Roth-Walter F., Jensen-Jarolim E. (2020). Dysbiosis of the gut and lung microbiome has a role in asthma. *Seminars in Immunopathology*.

[89] Bowerman K. L., Rehman S. F., Vaughan A. (2020). Disease-associated gut microbiome and metabolome changes in patients with chronic obstructive pulmonary disease. *Nature Communications*.

[90] Zhao Y., Liu Y., Li S. (2021). Role of lung and gut microbiota on lung cancer pathogenesis. *Journal of Cancer Research and Clinical Oncology*.

[91] Thibeault C., Suttorp N., Opitz B. (2021). The microbiota in pneumonia: from protection to predisposition. *Science Translational Medicine*.

[92] O'Dwyer D. N., Zhou X., Wilke C. A. (2018). Lung dysbiosis, inflammation, and injury in hematopoietic cell transplantation. *American Journal of Respiratory and Critical Care Medicine*.

[93] Garantziotis S., Palmer S. M., Snyder L. D. (2007). Alloimmune lung injury induced by local innate immune activation through inhaled lipopolysaccharide. *Transplantation*.

[94] Haak B. W., Littmann E. R., Chaubard J. L. (2018). Impact of gut colonization with butyrate-producing microbiota on respiratory viral infection following allo-HCT. *Blood*.

[95] Tiso M., Schechter A. N. (2015). Nitrate reduction to nitrite, nitric oxide and ammonia by gut bacteria under physiological conditions. *PLoS One*.

[96] Qureshi M. A., Girgis R. E., Dandapantula H. K., Abrams J., Soubani A. O. (2004). Increased exhaled nitric oxide following autologous peripheral hematopoietic stem-cell transplantation: a potential marker of idiopathic pneumonia syndrome. *Chest*.

[97] Bhalla K. S., Folz R. J. (2002). Idiopathic pneumonia syndrome after syngeneic bone marrow transplant in mice. *American Journal of Respiratory and Critical Care Medicine*.

[98] Sorge C., Pereboeva L., Westin E., Harris W. T., Kelly D. R., Goldman F. (2017). Pulmonary complications post hematopoietic stem cell transplant in dyskeratosis congenita: analysis of oxidative stress in lung fibroblasts. *Bone Marrow Transplantation*.

[99] Coppell J. A., Richardson P. G., Soiffer R. (2010). Hepatic veno-occlusive disease following stem cell transplantation: incidence, clinical course, and outcome. *Biology of Blood and Marrow Transplantation*.

[100] Kambham N., Higgins J. P., Sundram U., Troxell M. L. (2014). Hematopoietic stem cell transplantation: graft versus host disease and pathology of gastrointestinal tract, liver, and lung. *Advances in Anatomic Pathology*.

[101] Huang Z., Jing X., Sheng Y. (2019). (-)-Epicatechin attenuates hepatic sinusoidal obstruction syndrome by inhibiting liver oxidative and inflammatory injury. *Redox Biology*.

[102] Kanda J., Kawabata H., Chao N. J. (2011). Iron overload and allogeneic hematopoietic stem-cell transplantation. *Expert Review of Hematology*.

[103] Yeom M. Y., Kim Y. J., Chung N. G. (2015). Hepatic veno-occlusive disease may develop in secondary iron overloaded mice after allogeneic hematopoietic stem cell transplantation with total body irradiation. *Blood Research*.

[104] Huang Z., Sheng Y., Chen M., Hao Z., Hu F., Ji L. (2018). Liquiritigenin and liquiritin alleviated MCT-induced HSOS by activating Nrf2 antioxidative defense system. *Toxicology and Applied Pharmacology*.

[105] Periasamy S., Yang S. S., Chen S. Y., Chang C. C., Liu M. Y. (2013). Prophylactic sesame oil attenuates sinusoidal obstruction syndrome by inhibiting matrix metalloproteinase-9 and oxidative stress. *JPEN Journal of Parenteral and Enteral Nutrition*.

[106] Eissner G., Multhoff G., Holler E. (1998). Influence of bacterial endotoxin on the allogenicity of human endothelial cells. *Bone Marrow Transplantation*.

[107] Dayang E. Z., Plantinga J., Ter Ellen B., van Meurs M., Molema G., Moser J. (2019). Identification of LPS-activated endothelial subpopulations with distinct inflammatory phenotypes and regulatory signaling mechanisms. *Frontiers in Immunology*.

[108] Masetti R., Biagi E., Zama D. (2021). Early modifications of the gut microbiome in children with hepatic sinusoidal obstruction syndrome after hematopoietic stem cell transplantation. *Scientific Reports*.

[109] La Mura V., Pasarin M., Rodriguez-Vilarrupla A., Garcia-Pagan J. C., Bosch J., Abraldes J. G. (2014). Liver sinusoidal endothelial dysfunction after LPS administration: a role for inducible-nitric oxide synthase. *Journal of Hepatology*.

[110] Huycke M. M., Moore D., Joyce W. (2001). Extracellular superoxide production by Enterococcus faecalis requires demethylmenaquinone and is attenuated by functional terminal quinol oxidases. *Molecular Microbiology*.

[111] Luca M., Di Mauro M., Di Mauro M., Luca A. (2019). Gut microbiota in Alzheimer's disease, depression, and type 2 diabetes mellitus: the role of oxidative stress. *Oxidative Medicine and Cellular Longevity*.

[112] Tang J., Xu L., Zeng Y., Gong F. (2021). Effect of gut microbiota on LPS-induced acute lung injury by regulating the TLR4/NF-kB signaling pathway. *International Immunopharmacology*.

[113] Mottawea W., Chiang C. K., Muhlbauer M. (2016). Altered intestinal microbiota-host mitochondria crosstalk in new onset Crohn's disease. *Nature Communications*.

[114] Ostojic S. M. (2018). Inadequate production of H2 by gut microbiota and Parkinson disease. *Trends in Endocrinology and Metabolism*.

[115] Chistiakov D. A., Bobryshev Y. V., Kozarov E., Sobenin I. A., Orekhov A. N. (2015). Role of gut microbiota in the modulation of atherosclerosis-associated immune response. *Frontiers in Microbiology*.

[116] Wang X., Yang S., Li S. (2020). Aberrant gut microbiota alters host metabolome and impacts renal failure in humans and rodents. *Gut*.

[117] Handa O., Naito Y., Yoshikawa T. (2010). Helicobacter pylori: a ROS-inducing bacterial species in the stomach. *Inflammation Research*.

[118] Badgeley A., Anwar H., Modi K., Murphy P., Lakshmikuttyamma A. (2021). Effect of probiotics and gut microbiota on anti-cancer drugs: mechanistic perspectives. *Biochimica Et Biophysica Acta. Reviews on Cancer*.

[119] Engen P. A., Green S. J., Voigt R. M., Forsyth C. B., Keshavarzian A. (2015). The gastrointestinal microbiome: alcohol effects on the composition of intestinal microbiota. *Alcohol Research: Current Reviews*.

